# Promoting innovation in the objective structured teaching examination and feedback: clustering teachers to aid teaching evaluation

**DOI:** 10.1080/10872981.2019.1620544

**Published:** 2019-06-11

**Authors:** Ming-Chen Hsieh, Tsung-Ying Chen

**Affiliations:** a School of Medicine, Tzu Chi University, Hualien, Taiwan; b Department of Medical Education, Buddhist Tzu Chi General Hospital, Hualien, Taiwan

**Keywords:** Feedback, teaching observation, teaching reflection, faculty development

## Abstract

Problem: This study used the principles of feedback in a faculty development curriculum to enable clinical teachers to conduct objective structured teaching exercises for performance assessment. Intervention: the Flanders System of Interaction Analysis (FIA) was given to analysis of the data collected from a particular situation, to videotapes of simulated clinical teaching skills. Context: The Sparse K-Means clustering method, one-way ANOVA and post hoc tests were employed to cluster the most commonly used skills by teachers and compare the features of different clusters were then discussed. Outcome: The evaluation method employed in this study can be extended to more teaching methods and skills. Lessons Learned: that through teaching observation, clinical teaching skills and reflection teaching can be improved.

## Background

Medical education emphasizes providing feedback to learners of all levels. However, how feedback is given to clinical teachers and the content of this feedback are rarely evaluated [1]. Clinical teachers are crucial within medical education. If the faculty development curriculum provides teachers with understanding of their own teaching effectiveness, which makes them aware of their shortcomings and able to make modifications, their teaching effectiveness will increase.

## Literature review

When a clinician decides to become a teacher, faculty development units typically enroll them in various training courses [2]. However, what clinical teachers actually do at the bedside often differs greatly from the faculty training [3]. Some teachers are oblivious to these differences, whereas others can identify and address the gap. Therefore, when observing clinical teaching, which is the way of learning after completing the faculty development curriculum, clinical teachers should be encouraged to evaluate their own thoughts, learning, and teaching practices, thereby clarifying their nascent beliefs [4]. As teachers, they should thoroughly examine their role and their responsibilities to students but also their personal beliefs and practices in the context, becoming a model for the students, because clinical teachers are also learners [5].

## Teaching observation and feedback

Among all methods of collecting teaching performance data, the most common is teaching observation [6]. Teaching observation prompts changes in teachers’ instruction methods and can be conducted through individual or group classroom observation [7]. The strength of this approach is that it collects data directly related to teacher performance, and the observation records can be kept and used repeatedly. Its weakness, however, is that observation can disturb the teacher’s instruction [8]. Additionally, it does not detect sociological, psychological, political, and organizational factors relevant to the teacher [9]. Furthermore, being observed may cause the teacher to be more performative than usual [10].

Social interaction analysis has been promoted in the teaching context by socio-educational scholars and can be used to record and analyze teaching behavior under real teaching conditions, ultimately providing feedback to teachers on how they can improve their teaching and thus reducing the time and effort wasted by novice teachers [11]. Additionally, the analysis can serve as a reference during teacher evaluation. Among social interaction analysis methods, the most famous is the Flanders interaction analysis system (FIAS) [12]. Researchers can use the FIAS to conduct quantitative social interaction analysis. The FIAS is an observational tool used to classify the verbal behaviors of teachers and pupils as they interact in the classroom. Only verbal communication in the classroom is considered; nonverbal gestures are not taken into account. The basic assumption of the system is that in the classroom, a teacher’s verbal statements are consistent with his or her nonverbal gestures and overall behavior [13]. The system categorizes the verbal behaviours and the feedback of pupils in the classroom into 10 categories, each of which contains teachers’ verbal communications and nonverbal gestures, with which the system can be used to study teacher–student interaction [12].

Since 1992, some medical schools in the USA have developed objective structured teaching examinations (OSTEs) [14]. These are similar to the objective structured clinical examinations for evaluating medical students’ clinical skills in that an OSTE employs standardized students (SSs) to evaluate the teaching skills of clinical teachers or residents [15]. In each station of an OSTE, clinical scenarios are often constructed, such as bedside teaching, ambulatory care education, chart writing, and feedback giving. Clear objectives and standards are provided for the assessment, so the teaching skills of clinical teachers and residents can be effectively assessed [16]. Additionally, the SSs provide a highly realistic and low-risk simulated environment in which teachers can practice, experience new teaching techniques and behaviors, and receive direct feedback, which helps them adjust and improve their teaching [16].

On the basis of the aforementioned theories and background, this study used an OSTE for teaching observation. We videotaped teachers during their simulated clinical feedback teaching. The FIAS was used for teaching behavior classification. Teaching modes were used in structural clustering Sparse K-Means. Teachers of varying backgrounds were recruited and observed. We described and classified teachers’ behaviors to provide a reference for departments or stakeholder wishing to increase faculty development effectiveness.

## Methodology

This research was approved by the Institutional Review Board of Tzuchi Hospital (IRB 100–90). The research participants were 38 clinical teachers from 2011–2013. The participants received 40 hours of instruction on the Faculty Development Program in General Medicine, which included a 4-hour course on how to provide feedback. The content of the feedback section covered environment and atmosphere (creating a safe environment first), guidance and triggers (emotions are deduced), diagnoses and feedback clarification, improvement plans, application, retrospection, and confirmation (confirming messages). Lectures and both large- and small-group discussions were involved. An OSTE design was employed for summative evaluation. To attain objectivity, we analyzed one of the stations, feedback skills, which took approximately 15 minutes to complete, and analyzed the interaction of the 38 teachers with the SSs. The scenario in the teaching case regarding feedback skill was as follows. The SS (played by a resident) reported to the teacher about a case with acute gastric ulcer, bloody stool, and abdominal pain. The SS reported that the patient was being transferred to the intensive care unit and outlined the treatment procedure. Conflict had arisen because the family and medical team had different opinions. The teacher then had to give feedback and make suggestions on how to proceed.

## Research tools

### Introduction to the FIAS

The FIAS is a common system for observing the interactive behaviors of teachers and students in a classroom during teaching. The goal is to analyze teaching behavior and to record major teacher–student interaction events. Through understanding the effect of classroom interaction events, teachers can gain insight into and improve their teaching [12].

Flanders encoded all verbal events between teachers and students into a 10-category system. Recording is performed by observing the ongoing interactions in the classroom and recording them in the appropriate categories. The recording timeline of category number continued at the rate of 20–25 recordings per minutes for the entire 15-minute observation period. The behavior categories are displayed in Table 1.

**Table 1.** Flanders interaction analysis system verbal interaction categories.

Teacher talk	**Indirect influence (response)**	1. **Accept students’ feelings**: Use a nonmenacing manner to accept and clarify students’ attitudes or feelings tone. Students’ feelings may be positive or negative. This category also includes the prediction or recall of students’ feelings. (Accepts Feeling: Accepts and clarifies an attitude or feeling tone of a pupil in a non-threatening manner. Feeling may be positive or negative. Predicting and recalling feelings are included)。2. **Praise and encourage**: Praise and encourage students for their actions and behaviors. This category includes jokes that help release tension but are not hurtful. Examples include nodding or saying, ‘Um hmm’ or ‘Go on.’ (Praises or encourages: Praises or encourages action or behavior. Jokes that release tension, but not at the expense of another individual; nodding head saying um, hmm or go on are included.)。3.**Accept or use students’ ideas**: Clarify, expand, or develop ideas proposed by or thoughts of students. However, if a teacher presents more of his own opinions or thoughts, this is under Category 5. (Accepts or uses ideas of pupils. Clarifying, building or developing ideas suggested by a pupil. Teachers’ extensions of pupil ideas are included but as teacher brings more of his own ideas into play, shift to category five.)。
		4. **Ask questions**: On the basis of the teachers’ opinions or thoughts, students are asked questions on teaching content or steps and are expected to respond. (Asks questions: Asking a question about content or procedures; based on teacher ideas, with the intent that the pupil will answer.)。
	**Direct influence (initiation)**	5. **Lecture**: The teacher provides facts or opinions based on the teaching content. Teachers provide their own view or explanation, or they cite the view of an authority, but not a student. (Lecturing: Giving facts of opinions about content or procedures; expressing his own ideas, giving his own explanation or citing an authority other than a pupil.)。6. **Give direction**: The teacher provides direction or gives an order and expects the student to comply. This type of behavior requires students to comply. (Giving direction: Directions, commands or orders to which a student is expected to comply.)。7. **Criticize students or maintain authority**: The teacher makes a statement to try and alter a student’s behavior from unacceptable to acceptable. The students could be scolded. The teacher should explain why they take this approach. (Criticizing or justifying authority: statements intended to change pupil behaviour from non-acceptable to acceptable; bawling someone out; stating why the teacher is doing what he is doing; extreme self-references.)。
Pupil talk	**Indirect influence (Response)**	8. Student talk (response): Students talk in response to the teacher. The teacher requests a specific student responds, induces students to talk, or constructs a conversational situation. The students are not free to express their own thoughts. (Pupil-talk – response: Talk by pupils in response to teacher. Teacher initiates the contact or solicits pupil statement or structures the situation. Freedom to express own ideas is limited.)。
	**Direct influence (Initiation)**	9. Student talk (initiation): Students actively open a conversation. They express their thoughts and initiate new topics. They freely explain their own thoughts and ask inspiring questions. They do not follow convention. (Pupils-talk – initiation: Talk by pupils that they initiate. Expressing own ideas; initiating a new topic; freedom to develop opinions and a line of thought, like asking thought, like asking thoughtful questions; going beyond the existing structure.)。
**Silence**		10. Silence or confusion: There are pauses or short periods of silence or confusion, resulting in the observer unable to understand teacher–student communication. [Silence or confusion: Pauses, short periods of silence and periods of confusion in which communication cannot be understood by the observer.)。

Note: From 12, *Analyzing teaching behavior* (p.34]

### Analysis and statistical methods

Coding was conducted by two people. Videos of teaching sessions were recorded, and the coders then discussed the appropriate codes while watching the videos. To prevent the observers from making considerably different value judgments due to subjectivity, in addition to observer training, an interrater reliability test was conducted when the grading was performed. We randomly selected 10 videos of teaching sessions to evaluate the interrater reliability via calculating Kappa. The results of Kappa ranged from 0.73 to 0.90, showing that the scores awarded by the two observers for the 10 categories of teaching behavior were highly consistent. Because the participants who provided feedback were SSs acting according to a fixed scenario and with fixed dialogue, in the final analysis, the SSs’ reactions were excluded from the analysis. This study excluded the students’ responses and considered only 9 of the 10 FIAS verbal interaction categories for analysis. The categories were named F0–F8 (Table 2).

**Table 2.** Demographics (N = 38).

	N(%)
N	38
Sex	-
Male	28(73.7%)
Female	10(26.3%)
Department	-
Internal medicine	16(42.1%)
Surgery	3(7.9%)
Other	19(50.0%)
Work experience	-
Less experienced	20(52.6%)
Highly experienced	18(47.4%)
Hospital level	-
Medical center	20(52.6%)
Regional hospital	18(47.4%)

Data are presented as number and percentage.

We employed the sparse K-Means for analysis [17], it were adjusted for different FIAS categories and physician factors (age, sex, specialty, and hospital level). Clustering is the process of categorizing concrete or abstract objects into groups on the basis of their similarity. The standard k-means clustering is commonly used in data mining. For the number of clusters k, the algorithm is trained to arbitrarily select k item centroids and group each input item with its most similar centroid. The centroid in each cluster is then recalculated using the distance center of all the categorized centroids. The goal is to investigate the influence and grouping relationship of each cluster. One of the simplest unsupervised learning algorithms for solving clustering problem, K-means clustering has a simple and straightforward procedure for classifying a given data set into a certain number of clusters fixed a priori. These centroids should be selected intelligently because different locations would obtain different results. The optimal choice is to select centroids as far from each other as possible. The next step is to consider each point in the data set and identify which centroid it is nearest to [18]. Doing this for all points completes the first step and obtains the first groupage. The k centroids of each cluster are then recalculated. After the new k centroids have been obtained, the data points must be reassociated with their new closest centroid. This generates a loop. As the loop proceeds, the k centroids change location until their location becomes stationary. Sparse K-Means clustering is an established method of simultaneously excluding uninformative features and clustering the observations. After identifying clusters, we further compare the characteristics of different clusters. Statistical mean differences of continuous data between different clusters were analyzed with One-way analysis of variance (ANOVA) or Kruskal-Wallis test. The Bonferroni correction was used as post-hoc analysis. The Chi-square test or Fisher’s exact test were used to evaluate the association between two categorical<0.05. All analyses were performed using R (Version 3.5.0), the R-packages sparcl (Version 1.0.4), and SPSS software version 17.0 (SPSS Inc., Chicago, IL, USA).

## Results

**Figure 1.**
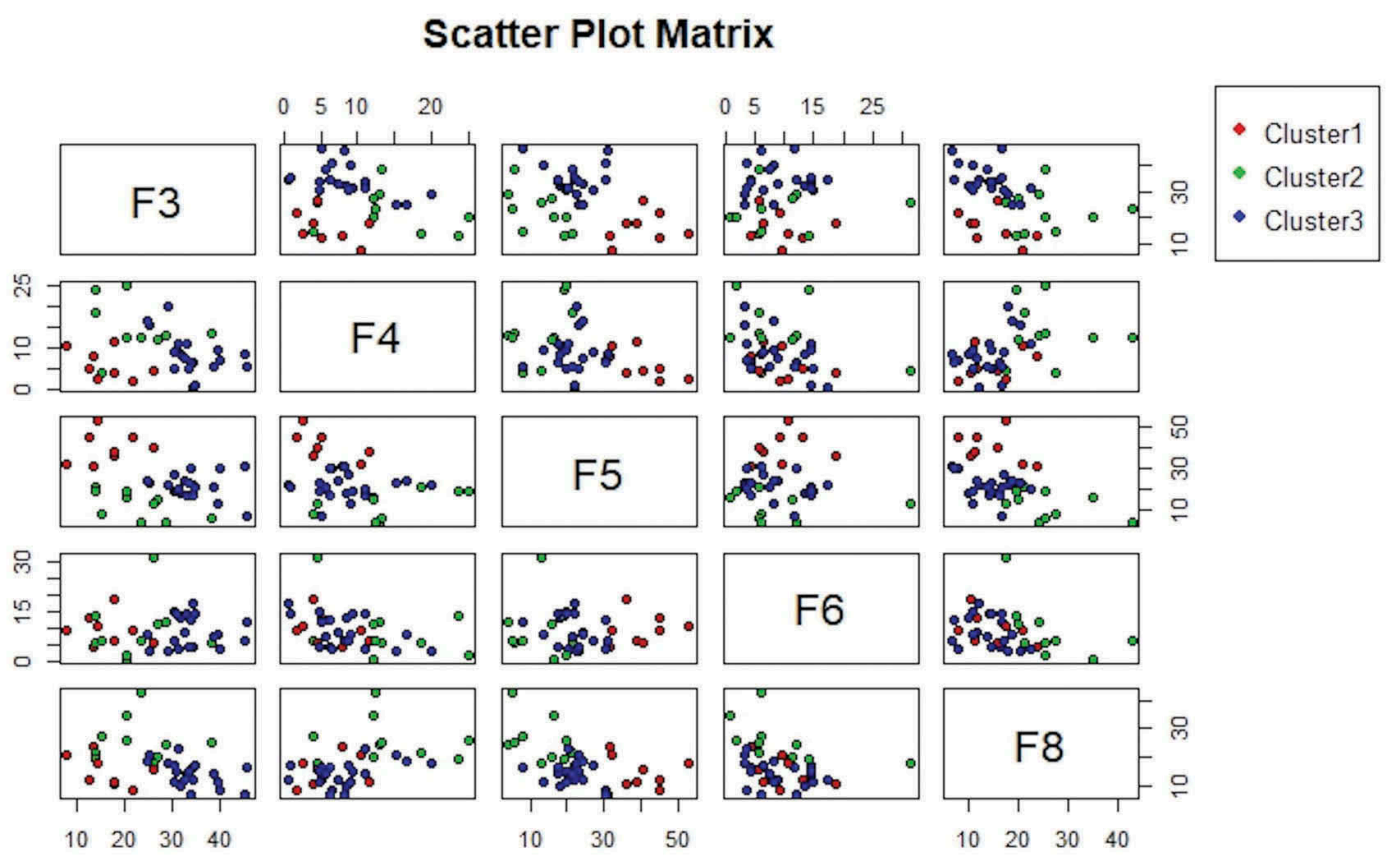
Visualization of cluster obtained using the Sparse K-Means algorithm.

**Figure 2.**
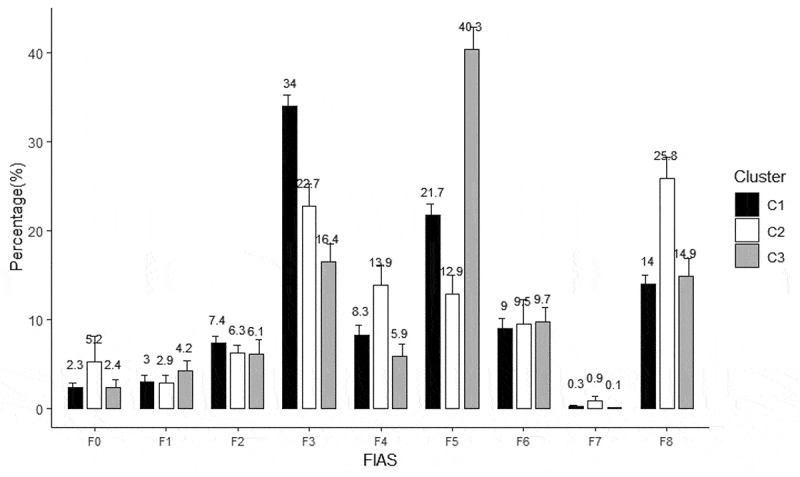
Flanders interaction analysis of the feedback scenario: C1 (N = 20): F3 (accepting or using ideas of students, response); C2 (N = 10): F4 (asking questions); C3 (N = 8): F5 (lecturing, initiation).

A total of 38 clinical teachers, 28 men and 10 women, participated in this study. Those with 5 or more years of teaching experience were considered highly experienced, and those with 4 years or fewer were considered less experienced. Slightly more than half of the participants were less experienced. The participants were from different levels of hospitals and from different departments (internal medicine department, 42.1%; surgery department, 7.9%; and other departments, 50%). The participants’ backgrounds are detailed in Table 3. We employed Sparse K-Means clustering to group the participants. Figure 1 presents the results of this clustering into three main groups (C1–C3). C1 (n = 20) comprised the participants who principally performed F3 category behaviors, which are indirect influences (responses) in which the ideas of students are accepted or used. C2 (n = 10) comprised the participants who principally performed F4 category behaviors, which are the asking of questions, and F8 category behaviors, which are pupil-talk–response interactions. C3 (n = 8) comprised the participants who principally performed F5 category behaviors, which are lecturing under direct influence (initiation). These three clusters have notably higher percentage of the teaching behavior via FIAS (Figure 2). No significant differences in the sex, department, experience, and group (hospital level) of these three groups of teachers were discovered.

**Table 3.** Characteristics of the three clusters.

	Cluster 1	Cluster 2	Cluster 3	Total	P-value	Post-hoc
N	17	13	8	38		
Sex	-	-	-	-	0.895	
Male	13(76.5%)	9(69.2%)	6(75.0%)	28(73.7%)		
Female	4(23.5%)	4(30.8%)	2(25.0%)	10(26.3%)		
Department	-	-	-	-	0.233	
Internal medicine	7(41.2%)	4(30.8%)	5(62.5%)	16(42.1%)		
Surgery	0(0.0%)	2(15.4%)	1(12.5%)	3(7.9%)		
Other	10(58.8%)	7(53.8%)	2(25.0%)	19(50.0%)		
Work experience	-	-	-	-	0.123	
Less experienced	10(58.8%)	4(30.8%)	6(75.0%)	20(52.6%)		
Highly experienced	7(41.2%)	9(69.2%)	2(25.0%)	18(47.4%)		
Hospital level	-	-	-	-	0.583	
Medical center	9(52.9%)	8(61.5%)	3(37.5%)	20(52.6%)		
Regional hospital	8(47.1%)	5(38.5%)	5(62.5%)	18(47.4%)		
F0. Silence or confusion	2.21 ± 2.39	4.65 ± 8.04	2.35 ± 2.48	3.07 ± 5.09	0.398	
F1. (Response): accept students’ feelings	3.45 ± 3.25	2.33 ± 2.62	4.21 ± 3.28	3.22 ± 3.05	0.371	
F2. (Response): praise and encourage	7.42 ± 3.79	6.44 ± 2.54	6.13 ± 4.47	6.81 ± 3.52	0.634	
F3. (Response): accept or use ideas of students	35.07 ± 5.25	23.95 ± 7.26	16.44 ± 5.77	27.34 ± 9.62	<0.001*	C1 > C2 > C3
F4. Ask questions	6.31 ± 2.84	15.17 ± 5.68	5.9 ± 3.62	9.26 ± 5.93	<0.001*	C2 > C1, C3
F5. (Initiation): lecture	21.06 ± 6.43	15.81 ± 7.35	40.3 ± 7.25	23.31 ± 11.4	<0.001*	C3 > C1, C2
F6. (Initiation): give direction	11.37 ± 6.75	6.31 ± 4.05	9.71 ± 4.61	9.29 ± 5.85	0.058	
F7. (Initiation): criticize or justify authority	0.27 ± 0.55	0.74 ± 1.21	0.09 ± 0.17	0.39 ± 0.83	0.160	
F8. (Response): student talk	12.84 ± 3.56	24.61 ± 7.06	14.88 ± 5.48	17.3 ± 7.52	<0.001*	C2 > C1, C3

Data are presented as number or mean ± standard deviation. *P < 0.05 was considered statistically significant.

## Discussion

The FIAS enabled clinical teachers to determine their performance on instruction, interaction, and coaching behavior of the teaching process after watching clips of their lessons [12]. If qualitative feedback can be provided together with timeline marking, more precise feedback and suggestions can be provided. Additionally, clinical teachers can objectively reflect on their teaching as well as gain inferences through comprehension, transformation, instruction, evaluation, reflection, and new comprehension, thereby positively affecting their future teaching.

This study discovered that after the teachers had finished the faculty development curriculum, when practicing the feedback skill in a simulated scenario, they could all ask questions to receive students’ opinions, listen intently, and express their own experience and thoughts, regardless of their experience and department. During the intense basic faculty development curriculum, the teaching effectiveness of the teachers improved. Through objective teaching observation, discussion, and recording, we determined how the teachers actually taught students on giving feedback. After completing the faculty development curriculum, the teachers could all demonstrate the following during practice teaching scenarios: understanding what the students were asking for help on; assisting them in setting learning tasks and goals; discussing the required skills with the students; clarifying the content of critical clinical handling principles, assessments, and evaluative discussions; and assisting students in strengthening their skills. These are the preliminary results of the faculty development curriculum.

## Conclusion and suggestions

Clinical teachers should all complete the teaching principle curriculum, which involves learning about theories and general rules in lectures, participating in small group discussions, observing the actions of role models in outstanding teaching cases, and scenario writing and amendment for actual teaching units. If the curriculum is completed by teachers formulating effective methods of summative evaluation for use in teaching practice and videotaping to provide feedback, clinical teachers will be able to organize the knowledge they acquire from the curriculum, and this knowledge will positively influence their teaching performance. To train excellent clinical teachers, time and effort are necessary to help those teachers develop mature teaching behavior. Faculty development units must be supportive of this process by providing more comprehensive course planning and following up on teachers’ progress.

## Limitations of the study

FIAS provides a simple way to collect and analyze teaching behavior automatically, yet it has some limitations. Firstly, some clinical interactive activities are not undertaken via interaction behavior (e.g., content expertise and interpersonal attributes), and thus they could not be perceived by FIAS so far. Secondly, the sample size was relatively small according to the most popular rule from Forman, which recommends a sample size of at least 2^k, where k is the number of variables used for clustering. Lastly, the extended dialogue between students and faculty cannot be analyzed at present. All these issues need to be addressed in future studies.
